# Social signals predict contemporary art prices better than visual features, particularly in emerging markets

**DOI:** 10.1038/s41598-024-60957-z

**Published:** 2024-05-21

**Authors:** Kangsan Lee, Jaehyuk Park, Sam Goree, David Crandall, Yong-Yeol Ahn

**Affiliations:** 1 Division of Social Science, New York University Abu Dhabi, Abu Dhabi, UAE; 2 School of Public Policy and Management, Korea Development Institute, Sejong-si, Republic of Korea; 3 Department of Computer Science, Stonehill College, Easton, MA 02357 USA; 4 Luddy School of Informatics, Computing, and Engineering, Indiana University, Bloomington, IN 47408 USA

**Keywords:** Computational science, Information technology

## Abstract

What determines the price of an artwork? This article leverages a comprehensive and novel dataset on art auctions of contemporary artists to examine the impact of social and visual features on the valuation of artworks across global markets. Our findings indicate that social signals allow us to predict the price of artwork exceptionally well, even approaching the professionals’ prediction accuracy, while the visual features play a marginal role. This pattern is especially pronounced in emerging markets, supporting the idea that social signals become more critical when it is more difficult to assess the quality. These results strongly support that the value of artwork is largely shaped by social factors, particularly in emerging markets where a stronger preference for “buying an artist” than “buying an artwork.” Additionally, our study shows that it is possible to boost experts’ performance, highlighting the potential benefits of human-machine models in uncertain or rapidly changing markets, where expert knowledge is limited.

## Introduction

Imagine yourself sitting at an obscure auction, bidding on an old portrait attributed to someone obscure. How much would you be willing to bid? Now, consider the same painting being auctioned at the Christie’s in New York, which is allegedly by Leonardo Da Vinci himself. How much would you be willing to pay? In 1958, the painting “Salvator Mundi”, previously attributed to someone in Da Vinci’s studio, was sold for a mere $60. However, after it was re-attributed to Da Vinci himself, the *same painting* was sold for $450 million at Christie’s New York in 2017, becoming the most expensive painting ever sold^1^. This extreme example highlights how an exceptionally renowned artist’s reputation can dwarf the innate qualities of the artwork. This tension between innate beauty and external fame has been two contrasting frames to examining arts. Is the value of artworks primarily based on the artist’s fame and reputation, or are such examples more exceptional than typical? To what extent can we assess the value of a piece without any knowledge of its intrinsic quality?

Assessing the value of unique cultural products, such as artwork, is notoriously difficult. As each work of art possesses unique and subjective aesthetic qualities, creating a standardized measure of its quality would be almost impossible. However, cultural valuation is not only an individual’s choice of artistic characteristics but also a highly socialized action^2–5^.

*Social constructionism* emphasizes the significance of social signals from the creators and distributors of art in determining its value^4,6–8^, rather than focusing on specific characteristics of the artwork itself. This can result in a weak connection between the quality of cultural products and their success in cultural markets^9^ and the important role of social networks in success^10^.

This is particularly evident in modern abstract or contemporary art, where there is less emphasis on figurative sophistication and a relatively shorter period of time for a consensus to emerge^11–13^. As a result, different social signals from both the artist and the market are considered significant determinants in the valuation process within the global art world^2,3,14–17^. In this perspective, the valuation may vary across markets based on the audiences’ level of understanding and shared values.

However, some argue that artistic quality has an unchanging value, as seen in *formalism*, a theory in the study of art and aesthetics. Formalist theories^18,19^ suggest that the value of an artwork is a result of its *artistic form*—the formal visual qualities of the artwork itself, such as color, line, composition, and shape, over its social, historical, or cultural context. Thus, the valuation process should remain consistent across different markets since the forms do not change^20,21^. Formalists believe that an artwork’s ability to express and convey meaning is the most essential factor in determining its value^5^. However, there has been no attempt to systematically quantify and compare the sizes of these two effects—social signals and artistic forms—on the market value of artworks.

This study aims to revisit and expand upon classical debates in valuation by quantitatively comparing the effectiveness of predicting market values based on the *visual* characteristics of the artwork versus *social (non-visual)* information about the artist and market. In addition, we further examine whether the impact of visual and social factors varies across established and emerging markets. Our aim is to determine whether social conditions, such as diverse audiences, influence the impact of social factors on cultural valuation, or if the form of artwork provides a universal experience to audiences.

Here, we use a novel dataset of 34,200 auction sales records, including images, artists’ attributes, and market information, encompassing 590 living contemporary artists spanning 17 years (1996–2012) across 23 countries. This comprehensive approach goes beyond the findings of previous studies on cultural valuations and its elusive quality metrics^10,22^ by analyzing the most systematically-collected dataset of contemporary arts and teasing out the contribution from visual and social features. We employ two machine learning models in predicting auction prices: one that utilizes visual features and the other that utilizes socioeconomic features. Then, we compare the contribution of visual versus social factors between established and emerging markets. The visual features are extracted from publicly available artwork thumbnails, while publicly available metadata for artists and market features are used for social features. We divide the transactions based on auction locations and select established markets, such as the USA, UK, France, and Germany, based on their historical significance and market size, and emerging markets, such as China, the Middle East, Brazil, Korea, and others. We concentrate on living contemporary artists and their artworks as they are more comparable to each other, but their shorter history compared to the works of old masters presents a greater challenge in their valuation^13,23–25^.

## Results

### Buying an artwork: computer vision analysis of visual features

**Figure 1:**
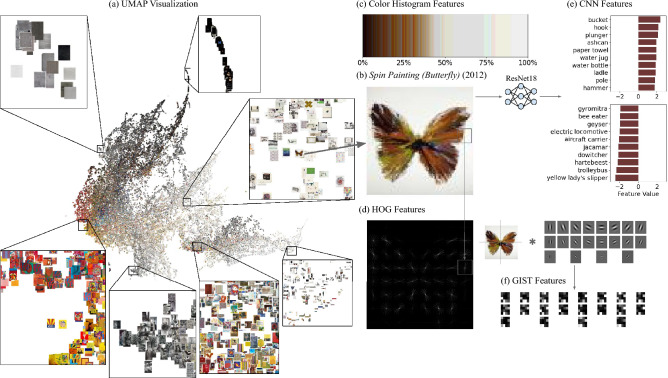
Our visual feature extraction process. (**a**) A UMAP dimensionality reduction of our total feature space, with several regions at high resolution showing organization according to visual characteristics. (**b**) One example, *Spin Painting (Butterfly)* (2009). (**c**) An area plot of the color histogram features. (**d**) HOG features, shown by the lightness of gradient lines in each region. (**e**) Bar graph of CNN features, which correspond to ImageNet classes. (**f**) Heatmap of GIST features for each Gabor filter shown above.

We first construct an XGBoost model predicting the prices of artwork only utilizing the visual features extracted from the artworks’ images. To extract the visual features, we apply multiple algorithms that are widely used in computer vision and pattern recognition fields, such as GIST^26^, HOG^27^, color histograms^28^, and deep learning-based features from a model trained on the ImageNet dataset^29–31^, while adding hand-tuned measures of colorfulness and complexity (presented in Fig. 1 and see “Materials and methods” for details). Although these features capture various characteristics of the visual forms that humans rely on to recognize objects and scenes, the total number of visual features generated by all algorithms is excessively large and redundant. Hence, we employ principal component analysis (PCA) to reduce the feature space for visual features. Subsequently, we utilize the XGBoost algorithm with these reduced 100 features (see   Materials and methods” for details) to predict the price of the artwork^32^.

As shown in Fig. 1, our visual features successfully capture the fundamental characteristics shared among artworks. The 2-dimensional representation of our dataset’s artworks using UMAP (Fig. 1a) provides insight into the contemporary art landscape by showcasing visual similarities, ranging from color composition to structural composition. Moreover, we test to determine whether the selected visual features could predict other characteristics of the artwork or artist, such as genre or gender. Employing the XGBoost classifier, we discovered that the visual features exhibited the ability to predict the genre of artwork; however, they proved to be insignificant in predicting other artist-level characteristics in contemporary arts (Table 2).

**Table 1:** Test set performance for computer vision analysis methods in predicting the price of an artwork, compared to a baseline model which predicts the mean of the training set. The visual features proved to be informative, with color histogram and GIST features being particularly useful. However, it was found that these two features were largely redundant with each other.

Model	Price (test set $R^2$)
Baseline (mean regressor)	− 0.002
All visual features	0.055
Color features	0.056
CNN features	0.009
HOG features	0.029
GIST features	0.048
Colorfulness and complexity	0.012

While the visual features extracted by our algorithm capture various aesthetic aspects of artworks, they have a limited impact on predicting the price of artworks. The results of our model, presented in Table 1 are measured in terms of $R^2$ value, which represents the variance of the estimated price explained by visual features. Our initial model, focusing solely on visual features, achieves the $R^2$ of 0.055, indicating a marginal level of explanatory power in determining the price of artworks. Although this model outperformed the mean prediction of the training set, it fell short by only explaining a mere 5.5% of the price variation.

**Table 2:** Prediction accuracy of a model based on visual features for several metadata characteristics. Each prediction is compared to a “dummy” classifier which always predicts the majority class (e.g. gender male, tier 1, country USA). Visual features are unhelpful to predict metadata characteristics other than genre. Please see Supplementary Materials for AUC and F1 scores.

Feature	Visual accuracy	“Dummy” accuracy
Gender	0.861842	0.861988
Education	0.809503	0.809941
Elite school	0.733626	0.733771
Genre	0.472076	0.321053

Our results suggest that, as one might expect, there may not be universal visual features that signal the “quality” or “value” of contemporary artwork, especially ones that can be captured by figurative distinctions from low-level visual feature extraction techniques.

### Buying an artist: metadata analysis of artists and markets

What would happen if we only use socioeconomic metadata, such as artist-related and market-related data, without any visual information about the artworks themselves? This approach—predicting the auction prices without any artwork-level features, especially visual ones—estimates to what extent the valuation is a social construct. Our features used in this model have mostly been tested in previous studies and they include thirty artist-level features such as age, gender, number of exhibitions, private/public acquisitions, and previous price levels, as well as eight market-level features, such as market size, art market growth, and the reputation of auction houses where each auction occurred (see “Materials and methods” for details).

**Figure 2:**
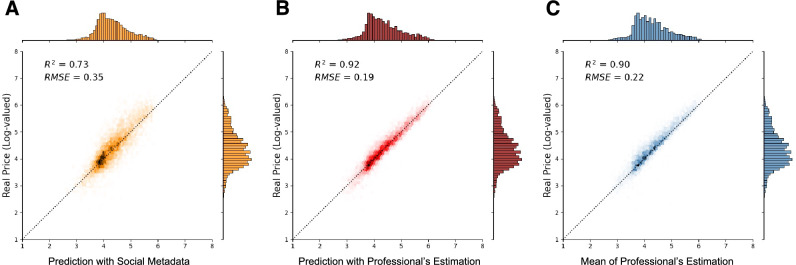
A machine learning model can predict the price of contemporary artwork without any content information and can improve upon the prediction of the auction houses. We show the correlation between the actual auction prices and the estimated prices with $R^2$ and Root Mean Square Error (RMSE) by (**A**) XGBoost model without professionals’ estimates, (**B**) XGBoost model with professionals’ estimates as an added feature, and (**C**) Arithmetic Mean of Professional’s Minimum and Maximum Estimates (base-10 logarithmic scale).

As a baseline, we first examine the estimated price ranges provided by art professionals in auction houses, taking into account all relevant factors including visual and social factors. These estimates are determined by auction houses that have exclusive access to extensive information about artists, artworks, and the markets and collectors^8,33^. The estimation provided by these professionals is a significant market signal because they *set expectations* about the value of an artwork. As a result, professional estimates may act as a self-fulfilling prophecy, particularly given the high valuation uncertainty^9^. Our findings indicate that these estimates are indeed highly predictive of the actual sales price, with about 90% of the variance explained (Fig. 2C).

In contrast to the results from our visual feature-based prediction (about 5.5% of variance explained), the XGBoost model that only uses social metadata surprisingly performs better (Fig. 2A), explaining about 73% of the variance. While this is still lower than the auction house’s estimates, it significantly outperforms existing models that typically range between 50 and 52%^2,3,24^ by a wide margin. Even considering previous studies highlighting the social construction of cultural valuation^6,7,11^, it is noteworthy that explaining 73% of the variation solely based on information about artists and markets is surprisingly high. One way to look at this result is that, because the model only uses information that exists prior to the creation of a given artwork, the model can predict 73% of the price variation *even before it is created*.

Furthermore, when we add the professionals’ estimates as a feature in our model along with the metadata, the model could improve the professionals’ estimates by explaining about 92% of the variance (Fig. 2B). This implies that while professionals’ estimates are highly informative and have a self-fulfilling effect, there is still scope for improvement through machine learning. It also suggests that professionals’ estimates can be further improved by leveraging only *publicly available information*.

**Figure 3:**
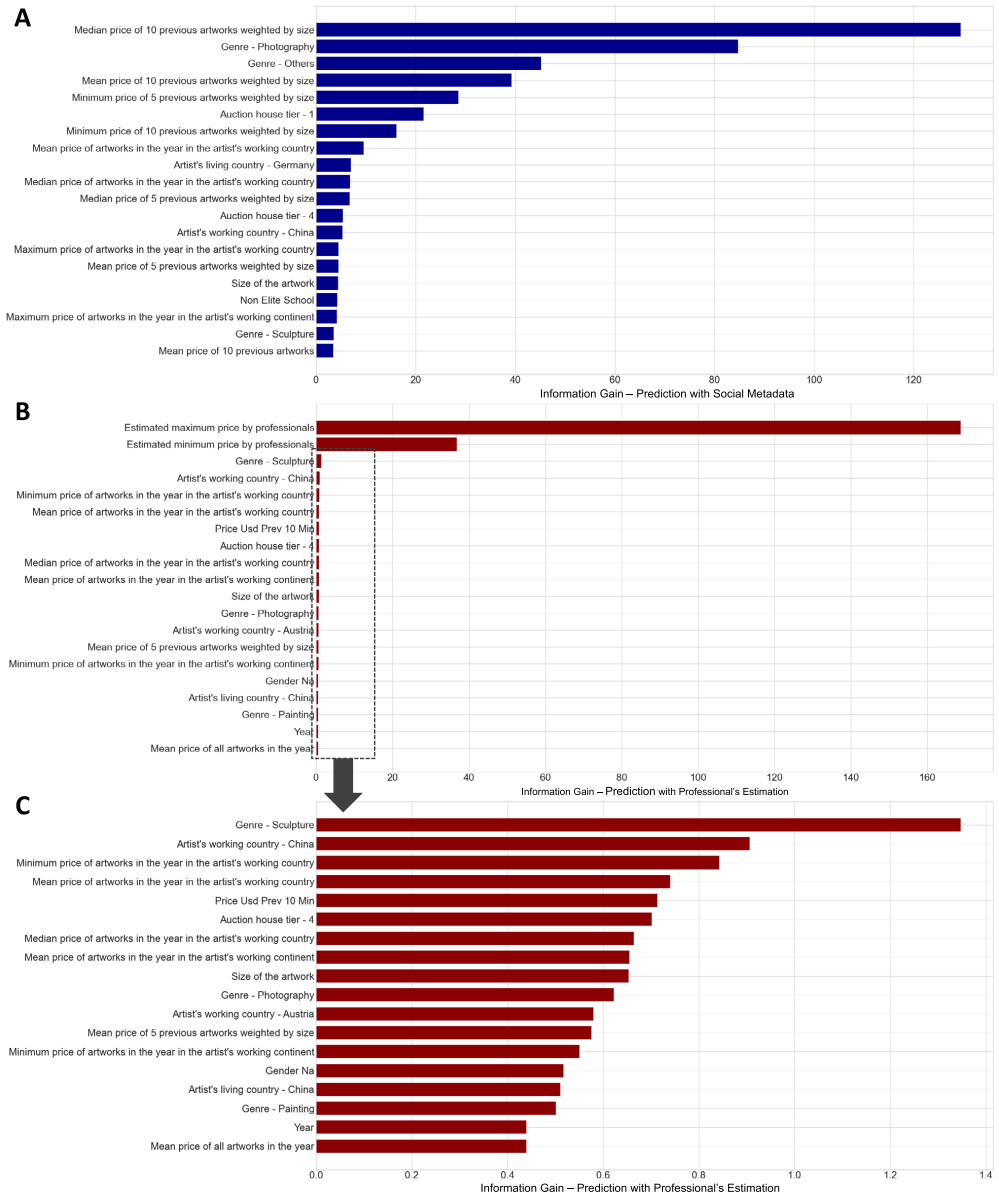
Top 20 features providing the largest information gain in estimating the auction price of an artwork. XGBoost model with social metadata only (**A**), with social metadata and professionals’ estimates overall (**B**), and the magnified version of the second model (**C**).

Then, how does our model predict the price so well? The top 20 features (see Fig. 3A) with the largest information gain in the stand-alone model without professional estimation align well with the three factors of the social construction theory of valuation. First, the most crucial set of features is the *past*—price levels of previous artworks sold by the same artist while controlling for the size and genres of artworks. For example, the average of the past 10 works is approximately 50% correlated with the price, and 49% correlated with the mean expert’s estimate (yielding an R-squared value of 0.25 compared to our final model’s 0.73). This is a higher rate than other similar features, such as the average of 10 random previous works which is approximately 44% correlated with both the price and the expert assessments. This finding provides strong evidence of the Matthew effect^34,35^ that the past market reputation of the artist and their existing recognition are critical in determining the price of new transactions^36,37^. As the contemporary art world increasingly relies on numeric forms of valuation as a result of commensuration and financialization^17,38^, previous price levels are commonly used to assess an artist’s relative status and make comparisons and reinforce the Matthew effect^2,39^. The significance of previous price levels in Fig. 3A, as captured by the mean and median prices of artworks by the same artist, underscores how auctioneers use past pricing patterns to estimate price ranges^17^.

Second, the machine learning model highlights the significance of the auction venue, specifically the tier of the auction house. This finding aligns with Bourdieu’s theory of cultural capital and the conversion of cultural value into economic capital^7,40^. According to this perspective, collectors of contemporary art are well aware of the high-status and influential auction houses, which are perceived as a significant pricing signal^13,17^. Each auction house has its bidding ratios and a different price of return on similar artworks^41^, leading artists to compete for inclusion in the top auction houses in major cities. Such social hierarchy and prestige of auction houses can be self-perpetuating and can influence the valuation. The importance of venues in artistic performance is also evident in primary markets, as exhibition networks project the social and artistic reputation of artists^10^.

Finally, the remaining features in Fig. 3A and the most important features in Fig. 3C, which are the rest of the features except the professional price estimations in Fig. 3B, are more closely related to *where* the auction occurred, reflecting the puzzle (and complexity) posed by the recent expansion of the global art market. The contemporary art market is currently experiencing unprecedented and rapid expansion in both Asian and global markets^17,25^, Tier 4 art auctions—outside of major auctions in the US or Europe as well as those in China—draw attention to the significance of transaction locations. This suggests that the sales location is an important factor that cannot be explained by professional estimates alone. This highlights the emergence of new markets in the global art landscape where the new rules are still being shaped (Fig. 3C).

The rapid global expansion of the art market and the emergence of new art markets (i.e., China and the Middle East) have created greater interconnectivity across borders^42^, but have also presented challenges to the traditional art world in terms of valuation and consumption^43^. Recent studies highlighted not only the ongoing process of conventional gatekeeping in new markets by reproducing the existing norms and conventions of valuation but also the traditional professionals’ lack of understanding about these new markets^17,44,45^.

### Between established and emerging art markets

Building on our finding of the significance of auction locations in predicting artwork prices, we conduct another test to evaluate the predictability of our models, both visual and metadata, and professional estimation across different markets. We categorized the auction locations into two groups: established and emerging markets. Established markets comprise the primary art hubs, such as the United States, the United Kingdom, France, and Germany, due to their historical importance and the size of their art markets. Emerging markets include all other countries, except those in Europe, such as China, Brazil, Korea, and Qatar.

We first expect that there will be less variation in the predictions of visual features across markets, given the consistent visual quality. For social features, however, we anticipate greater variation between established and emerging markets, as distinct audiences across different countries may rely on shared social signals to varying degrees. In addition, we aim to determine whether our model can more accurately predict price patterns in emerging markets, despite their high uncertainty. This depends on whether the socially constructed valuation process is stronger in these new markets, as previous studies have suggested^16,46^.

**Figure 4:**
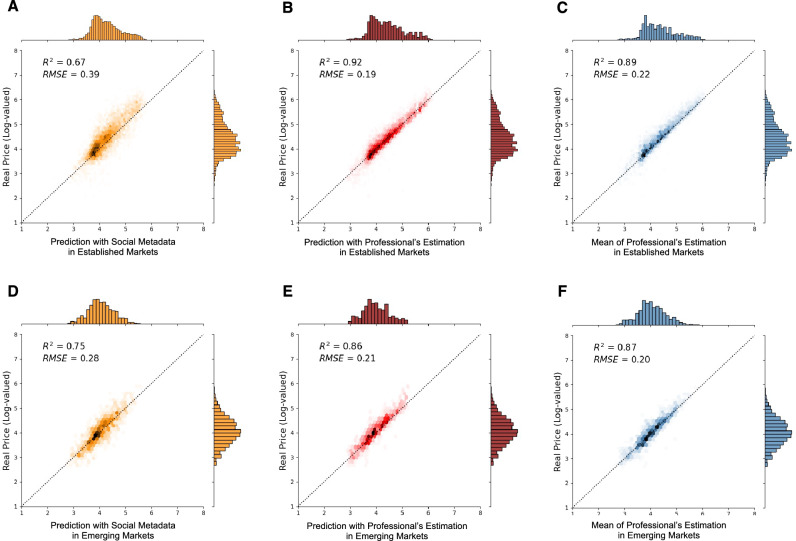
Accuracy comparisons of our XGBoost models without professionals’ estimations (**A**,**D**), our models with professionals’ estimations (**B**,**E**), and professionals’ estimations (**C**,**F**) between established markets (USA, UK, France, and Germany) and emerging markets (otherwise). While professionals predict the artworks sold in established markets more accurately than those in emerging markets ($R^2$ = 0.89 vs. 0.87), our model without professionals’ estimations predicts the artworks sold in emerging markets better than those in established markets ($R^2$ = 0.75 vs. 0.67). Together with professional estimation, the result shows a better prediction in the established market.

Initially, we verify that price predictions based on visual forms continue to be marginally significant across markets, thereby explaining the marginal but consistent interpretation of artistic forms across various markets as presented in Table 3. The difference in prediction for visual features between established and emerging markets is negligible compared to that of metadata features, which show stronger but different predictive power.

**Table 3:** Performance of visual and metadata features split by established and emerging markets. As we anticipated, the lack of significance of visual features remains consistent across markets.

Market	Model	Price (test set $R^2$)
Established	Baseline (mean regressor)	− 0.002
	Visual features	0.053
	Metadata (no prof. est.)	0.667
	Metadata (with prof. est.)	0.916
Emerging	Baseline (mean regressor)	− 0.002
	Visual features	0.056
	Metadata (no prof. est.)	0.750
	Metadata (with prof. est.)	0.859

Figure 4 demonstrates the predicted variance comparisons across three sets of models, the XGBoost model with social metadata only, the XGBoost model incorporating professional estimations as additional features, and the model comprising solely of professionals’ estimations. Notably, these models were tested on separate samples, established markets and emerging markets.

The results show that while the professional estimation predicts better in established markets, our machine-learning model offers increased explanatory power in emerging markets. This result supports the social constructionism finding in the previous tests.

The less effectiveness of professional estimation in emerging markets is attributed to the slower acquisition of expertise by professionals. In emerging markets, this process is not yet fully established, owing to the distinctiveness of new audiences and the level of market maturity. For example, during the Contemporary Spring auction in 2015, Shanghai Christie’s auctioneers noted that “Many people here are fairly new to art auctions and we still need to learn about them and create a relationship to understand what they want and to return” (Author interview, April 24, 2015). Professionals tend to build relationships with local collectors and artists over an extended period, facilitating a shared understanding of the value of artworks^11^. While professionals possess both formal and tacit knowledge and experience of established market conditions^47^, they are still in the process of familiarizing themselves with new audiences and engaging with them to attract more collectors in emerging markets^17^.

In addition, the results from emerging markets exhibit that socially constructed consumption is a major driver of its variation, which leads to a strong preference for buying artists. Collectors in these markets have relatively limited experience and knowledge about the subtle variation across the artworks of an artist, leading to a greater dependence on social signals as a means of coping with market uncertainty. This is due to the fact that professional guidance regarding artwork-based distinctions is not yet fully elaborated and shared in emerging markets.

In other words, established markets exhibit more variance, which cannot be solely explained by publicly available information about artists and markets. Through their extensive experience and close relationships with professionals, collectors in established markets not only comprehend differences in the prestige among artists but also gain access to the knowledge of subtle distinctions across artworks, even those created by the same artist. They often use this knowledge to express their distinctive cultural capital and distinguish themselves from other collectors^47^. Having said that, established markets are better equipped to appreciate and acknowledge the distinctiveness of artworks, and to recognize differences across artworks of the same artist aligned with professional valuations. In contrast, emerging markets place greater emphasis on social prestige at both the artist and market levels, which can be captured more efficiently, faster, and effectively using machine learning algorithms.

## Materials and Methods

### Data Collection

We selected the top 1,000 living contemporary artists based on their rankings by ArtFacts.net in January 2015, which was the time when we collected our data. It is worth noting that our focus in this study is on living contemporary artists for two main reasons. Firstly, if we were to include deceased artists, there would be a significant variation in terms of: a) the age of their artwork (i.e. how much time has passed since their death); b) random historical events; and c) the social consensus, which could all affect the price of their artwork at any given point. Secondly, there is a high probability of a reduction in uncertainty when artists die, as their impact on the market dynamics of their works is restricted or removed. Among art connoisseurs, the death of an artist restricts the supply of new artwork to the markets, allowing for the stabilization of the artist’s identity and prestige^3^. Over time, there tends to be a common understanding about the deceased artist, which generally increases their value. However, it should be noted that this generalization may not hold true for relatively unsuccessful artists, for whom the information may be limited and inaccessible.

After selecting the famous living artists, we collected relevant information manually from their webpages, as well as publicly available popular art databases such as ArtNet.com, ArtPrice.com, Blouin Art Index, and Artfacts.net. We also collected auction sales data between 1996 and 2014 from Blouin Art Index and validated it by cross-checking with publicly available auction house data at the time, such as Christie’s and Sotheby’s. As art auction prices serve as a proxy for the market value of an artwork^3,41,33,23^, we excluded artists who did not sell any artworks at auction houses, as well as those without publicly available information on their careers, such as exhibition records or artistic recognition awards.

At the same time, to empirically test the importance of visual qualities in artworks, we used publicly available thumbnail images of artwork associated with our auction records. High-resolution digital images of artwork are not usually available to the public due to the risk of unauthorized reproduction. Therefore, we collected publicly available thumbnail images. The availability of images online excluded 20,116 records, which dropped many of the transactions in 2013–14 when the images were not available. To avoid a highly skewed sample of later data, we cut off our sample in 2012. While some may argue that using low-resolution images leads to a loss of crucial visual information, it’s important to note that no photographic resolution is ever high enough to fully capture the artistic content of a physical artifact, and different resolutions will result in different digital representations. Therefore, we utilized the largest amount of publicly available data with a consistent resolution.

As a result, our final dataset includes 34,200 records of artwork of 590 living contemporary artists across 23 countries, spanning 17 years (1996 to 2012), including images of the art, artist-related features, and global art market features. For established and emerging market samples, the former includes 29,853 records across four countries (USA, UK, France, and Germany) while the latter includes 4347 records from 19 countries.

Considering the temporal structure of our dataset, the market transactions of artworks at auctions, we split the dataset into a training set with the earlier 27,360 transaction records (80% of our records) and a test set (the later 20%, 6840 transactions), which enable us to respect the temporal ordering and avoid data-leakage about future trends. This split simulates predictions about auctions between May 2011 and December 2012 using a model trained in May 2011 based on the past 15 years of data. For established and emerging markets, we maintain the same cutoff date, yielding training sets of 24,163 and 3197 works, and test sets of 5690 and 1150, respectively.

The datasets generated and analyzed during the current study are available in the Figshare repository with the accession number(s): 10.6084/m9.figshare.24746268.

### Feature Selection—Metadata

Our model includes eight market-level features—such as minimum, mean, median, and maximum prices of all other auctions in the continent and country where the target artwork is being auctioned, as well as auction house tier—and thirty artist-level features, which are listed in Table 4. For artwork, we only considered size and genre information. While the size can significantly modulate the price, we can safely assume that it does not inform us about the objective quality of the artwork. Thus, we standardized the prices by size to control for the potential price differences based on the size of each artwork. Larger pieces of artwork usually cost more due to the quantity of material involved (canvas, inks, paints, etc), but this does not necessarily reflect the quality of the artwork as well.

**Table 4:** List of features included in our social metadata analysis model without artworks’ information and professional’s estimation, with a brief description of the features.

Feature Name	Description
*Width, Height, and Square-inch size*	Width, height, and square inch size of an artwork
*Genre - f{Genre Name}*	Genre of an artwork (Painting, Print, Photograph, Drawing and Others)
*Age*	Age of an artist
*Gender*	Gender of an artist
*Education*	Education level of an artist (Domestic/Abroad, Elite School or not, and Degree)
*Price - f {Mean / Median / Maximum}*	Mean / Maximum / Minimum / Median price of the artworks by an artist during the year when auction is occurred.
*{Minimum / Mean / Median} price of {5 / 10} artworks*	Minimum / mean / median price of the artworks by an artist during the last five and ten transactions
*{Minimum / Mean / Median} price of {5 / 10} artworks weighted by size*	Size-standardized minimum / mean / median price of the artworks by an artist during the last five and ten transactions
*Award*	Records of art awards won by artists
*Biennial*	Records of participation in art fair or biennial
*Ranking*	Global artistic ranking of the artist as determined from U.S. and European art sources
*Solo and Group Shows*	Number of solo and group exhibitions for an artist until the auction year, respectively
*Match - Genre*	Whether an artwork is identified as part of the major genre of the artist
*Match - Country*	Whether an artwork is sold in the working country of its artist
*Private Acquisition*	Number of private acquisitions of artist works by individual collectors
*Public Acquisition*	Number of public acquisitions by museums
*Artist’s working country - {Country Name}*	Country where an artist is mainly working
*Artist’s living country - {Country Name}*	Country where the artist is living
*Auction house tier - {1/2/3/4}*	Tier of auction houses are coded based on the market dominance (the frequency and the volume of auctions) and the reputation of the auction house. Tier 1 includes global auction houses in major cities, such as Christie New York, Sotheby London, and de Pury New York, and the rest of international branches from the same global auction houses are grouped as tier 3 while the other major stand-alone auction houses were coded as tier 2 such as Dorotheum, Artcurial, and Kunsthaus Lempertz. Tier 4 is for the rest, non-major auction houses.
*Year*	Year of auction
*{Continent / Country} - {Minimum /Mean / Median / Maximum}*	Global art market size and growth based on the mean/maximum/median price levels across 26 countries in six continents for the year of auction
*Estimated {minimum / maximum} price by professionals*	Minimum and maximum prices of an artwork estimated by professionals

### Feature Selection—Image

We first use a vector of 8971 features to describe the art images (960 GIST features, 2915 HOG features, 4096 color histogram features, and 1000 CNN features). While we explored end to end deep learning-based methods for price prediction, their tendency to overfit led to poor validation set performance, which makes us focus our inquiry on interpretable visual features. In this section, we describe how these features are computed and how they roughly capture four key formal characteristics of art: composition, shape, color, and recognizable objects.

- *Composition*: GIST features^26^ are a classical computer vision technique for scene classification that is constructed using multiple scales and orientations of Gabor filters. They coarsely capture the composition of a scene by identifying directional frequency content, such as the horizon in landscapes.
- *Shape*: HOG features^27^ are another classical computer vision technique originally introduced for human detection, but then used for shape detection more broadly. They measure the histogram of oriented gradients, a circular histogram of all the directions of edges, for each region in the image.
- *Color*: Color histogram features are the number of pixels with each RGB color value, binned into 16 ranges per dimension (i.e. yielding 4096 bins). The captured color histograms are useful for identifying the dominant colors used in a work and are also widely used for content-based image retrieval (e.g.^28^, ^48^).. These features capture color information.
- *Objects*: CNN features use the final layer of a deep convolutional neural network image classifier, in this case ResNet18^31^, trained on the ImageNet dataset^29^. Since ImageNet has 1000 classes, there are 1000 features corresponding to the image classes. While the image classes are defined based on photographic data which is quite different from artwork thumbnails, the latent space learned by such a model can still be useful for prediction.

We observe that these features, especially the color histogram, are quite sparse and often highly correlated. To reduce the redundant dimensionality and avoid overfitting, we perform principal component analysis, keeping 100 visual components for full dataset analysis and 10 components for analysis when split by market sector, to avoid overfitting.

In addition to these relatively low-level visual features which correspond to specific characteristics like edge directions and colors, we compute two high-level features: predictions of perceived colorfulness and complexity. We predict perceived colorfulness using the average distance from each pixel to the image mean on the a-b plane in Lab color space (adapted from^49^). We also predict perceived complexity using the average value of a Canny edge detector on the saturation channel in HSV color space^50,51^. We find that these features are both useful for visualizing an artist’s collected work, as well as informative to the model.

### Extreme Gradient Boosting (XGBoost) Regressor

We use Extreme Gradient Boosting (XGBoost) regressor for our prediction models, which has won various machine learning competitions^32^ as well as has been applied to a wide range of applications from predicting mortality of patients^52,53^ to evaluating personal credit^54^. XGBoost is a high-performance machine learning algorithm based on the gradient-boosting decision tree. XGBoost can efficiently construct boosted trees and run in parallel—either regression trees or classification trees—and optimize the value of the objective function across the trees.

The major advantage of XGBoost is its scalability in diverse situations with a fast learning process^32^. The model works by combining a set of weaker machine-learning algorithms to obtain an improved machine-learning algorithm as a whole^55^. Also, XGBoost’s recursive tree-based decision system provides great interpretability potential. Although the internal model mechanisms of black-box modeling strategies are still difficult to interpret in general, the importance of each individual feature can be determined by its accumulated use in each decision step in trees. The metric calculating the relative importance of each feature is valuable to estimate features that are the most enhancing the outcomes of a model, especially when they are related to meaningful valuation parameters.

For each dataset and feature set, we perform hyperparameter search over maximum tree depth and learning rate using a validation set with size equal to the corresponding test set sampled at random from the training set.

## Discussion

The proliferation of “big data” and enhanced computational capabilities for data analysis have enabled the empirical reassessment of classical questions regarding human behavior and culture. One such debate is the valuation of art, where advocates of the artistic forms assert that the worth of an artwork is determined by its significant visual forms to individual viewers, whereas social constructionists posit that the value of a unique object is influenced by collective social and cultural processes. After analyzing the relative importance of social and visual features in predicting auction prices of artworks, the findings demonstrate that social factors of artists and markets play a significantly large role in the valuation of contemporary artworks rather than visual forms. This effect is particularly pronounced in emerging markets relative to established ones.

It might not be surprising that visual features have limited influence on the valuation of contemporary art, given that the figurative differentiation of artworks is less important in this context. There is always a possibility that a better model with novel features will be able to predict the price better. Yet, the fact that the other model, with a mutually exclusive set of features, approaches the accuracy of expert prediction, reaching 75% of price variation explained in emerging markets, sets a tight upper limit on how much the visual features (or the “beauty”) of the artwork can explain the price. It is also important to note that this prediction was solely based on publicly accessible data, without any additional information about the artwork itself or insider knowledge.

This discovery underscores the notion that cultural consumption is primarily a socialized activity reinforced by social mechanisms^6^ including the Matthew effects of existing recognition^35^, the social status of intermediaries (auction houses), and geographical locations of cultural markets.

Furthermore, the discrepancy between machine learning and professional estimation in established and emerging markets exhibits how socially constructed consumption exerts a significant influence on emerging market variation, leading to a stronger preference for *“buying artists”* and their prestige.

Lastly, although domain expertise remains critical in cultural industries, especially in established markets, as evidenced by our findings, this study’s results underscore the potential benefit of the human–machine learning model, integrating machine learning methods with expert knowledge to better understand the complex dynamics of cultural consumption. This approach has particular relevance for emerging markets, where professional expertise is less established.

However, our methodology does present certain limitations that warrant further exploration. First, since XGBoost models employ an extensive ensemble of decision trees, each individual tree determines price estimates based on a specific set of features. Consequently, the impact of each feature doesn’t follow a linear path but rather varies depending on the values of other features within the same tree. It is important to highlight that such flexibility and nonlinearity of certain features also reflect the complexities inherent in real-world valuation processes. In these contexts, the influence of one factor is often interrelated with, and affected by, other associated factors. However, our analysis of individual-level tendencies using the SHAP values^56^ reveals that the directional impacts of our main features align consistently with findings from existing research. This correlation substantiates the reliability and coherence of our model (Supplementary Materials Fig. 2).

Second, our visual analysis, while not statistically significant, does not rule out the possibility that additional visual aspects can be significant to the valuation process in different ways. For example, a recent study on artworks’ strokes shows its importance for attribution and authentication^57^. The concept of the “aesthetic gap,” which refers to the disparity between information that can be directly derived from the image itself and information that requires inferring the emotional state and contextualized understanding of the image from a human viewer, has been discussed in the computer vision and multimedia retrieval communities^58,59^. It is possible that different approaches may capture the constructed meaning in contemporary art behind the various layers of an artist’s unique style, which can explain the relationship between visual features and valuations. Such investigations could result in better predictive performance on prices or other career patterns of contemporary artists.

Third, our prediction was solely based on publicly accessible data, without any data about insider knowledge. However, it is widely acknowledged that insider knowledge and informal relationships play a key role in the valuation of artworks, especially during the early stages of an artist’s career, influencing the pricing of their artworks^11,60^. This becomes particularly evident when there is a scarcity of prior transactions. As such, future research should focus on addressing the challenge of limited observations among emerging artists in nascent markets, aiming to explore the intricate connection between insider knowledge and the interpretation of status signals and artistic styles. This will significantly extend our understanding of cultural valuation in general, especially within the context of early-career producers and emerging markets.

Finally, one could argue that it is impossible to separate the influence of artist, artwork, and art market on price, as artwork-specific information is already included, for example, in the price history of artists, and artist identity can sometimes be determined based on visual information. While such leakage is possible, artworks are generally considered artistically unique in every way. The clear performance difference between visual and metadata models indicates that the information contained in the social metadata and visual features are mostly distinct.

Nevertheless, our study demonstrates a stark difference between the information conveyed by the visual and social aspects of artworks in influencing their market value, reinforcing the idea that the market value of cultural products is mostly determined by how they are perceived by others at a producer level, rather than their inherent characteristics at an object level. Our findings also suggest that audiences in emerging markets follow these patterns, thus making it easier to predict their market valuations using machine learning approaches.

### Supplementary Information

Supplementary Information.

## Data Availability

The datasets generated and analyzed during the current study are available in the Figshare repository with the accession number(s): 10.6084/m9.figshare.24746268.
