# Supplementary material for: Social signals predict contemporary art prices better than visual features, particularly in emerging markets
Source: Sci Rep. 2024 May 21;14:11615. doi: 10.1038/s41598-024-60957-z (PMC11109285; doi:10.1038/s41598-024-60957-z)
Supplement: Supplementary file 1 — Supplementary Information. [file 41598_2024_60957_MOESM1_ESM.pdf]

Supplementary Information for
"Social signals predict contemporary art prices better than visual features, particularly in emerging markets"

Kangsan Lee,[1*†] **Jaehyuk Park,**[2*] **Sam Goree,**[3*] **David Crandall,**[4] **Yong-Yeol Ahn**[4]

[1]Division of Social Science, New York University Abu Dhabi, Abu Dhabi, UAE

[2]School of Public Policy and Management, Korea Development Institute, Sejong-si, Republic of Korea

[3]Department of Computer Science, Stonehill College, Easton MA, 02357, USA

[4]Luddy School of Informatics, Computing, and Engineering, Indiana University, Bloomington, IN 47408, USA

[*]Kangsan Lee, Jaehyuk Park, and Sam Goree contributed equally.

[†]To whom correspondence should be addressed; E-mail:ks.lee@nyu.edu

1 Figures

1.1 Descriptive trends of the artworks in our dataset

The number of artwork sold on auctions in our sample increased steadily over time (Fig. S1(A)). The price distribution of the artwork is close to log-normal, ranging from \$9 to \$3,248,105 after size standardization (Fig. S1(B)). The total sales value reaches \$2,074,998,696 for our final sample, 36,549 works of art, which is about 42% of the total sales of the contemporary art sector in 2012, according to the art sales reports (Tefaf art market report 2015 Art Basel and UBS report 2017). This is a significantly large and representative sample since the total sales include primary markets, dealer and gallery sales and private sales, while our data is only containing auction sales. Geographically, as illustrated in Fig. S1(C), the auction locations in our dataset are spread across the world, including both famous countries in the art market, such as the United States and Western European countries, and emerging countries in the market, for example China, India, and Australia.

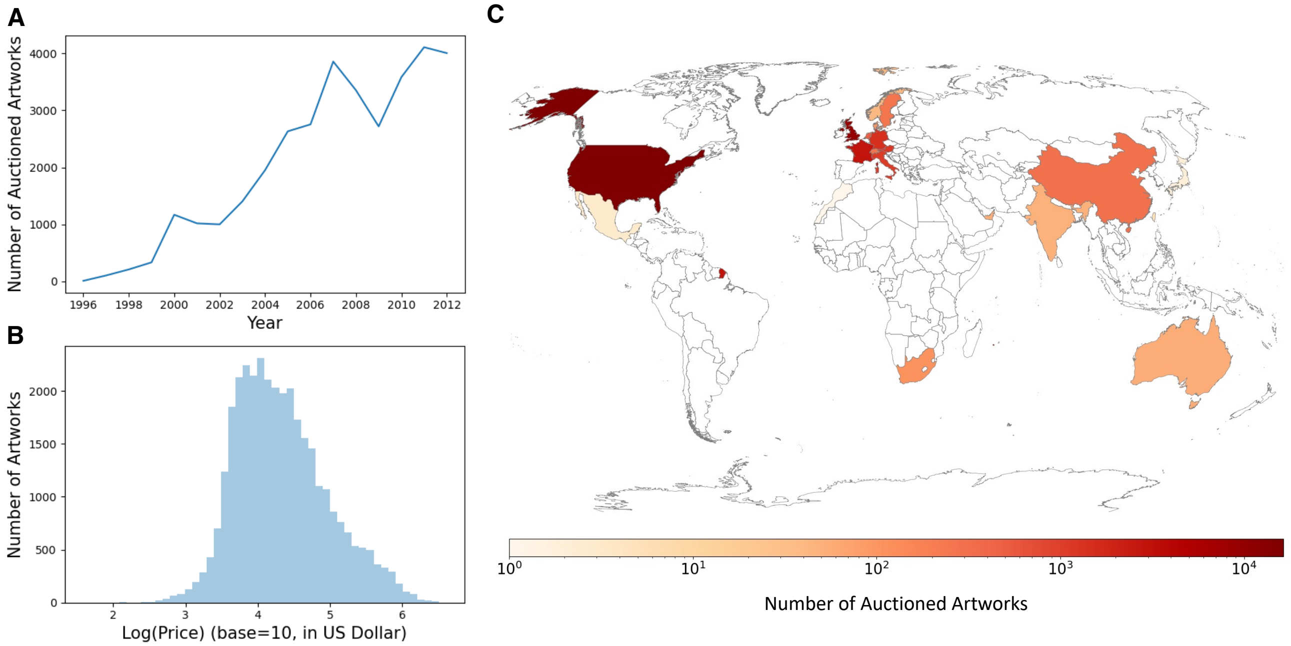

Figure S1. The distributions of artwork in our dataset across (A) time, (B) price, and (C) geography. Overall, the sale of the selected artists increased over time except during the 2008-2009 global economic crisis. (C) instances the globalization of art markets showing the coverage of various global markets of our sample artists, with increased sales in China, India, Brazil, and Australia.

1.2 SHAP values

2 Tables

2.1 Additional Classification Metrics

Table S1 shows AUC and F1 scores for classification problems reported in the main text. These metrics are more reliable than accuracy for imbalanced classification problems, like these. The AUC, the area under the receiver operator characteristic curve, for binary classification problems can be interpreted as the probability that an example of one class will score higher than an

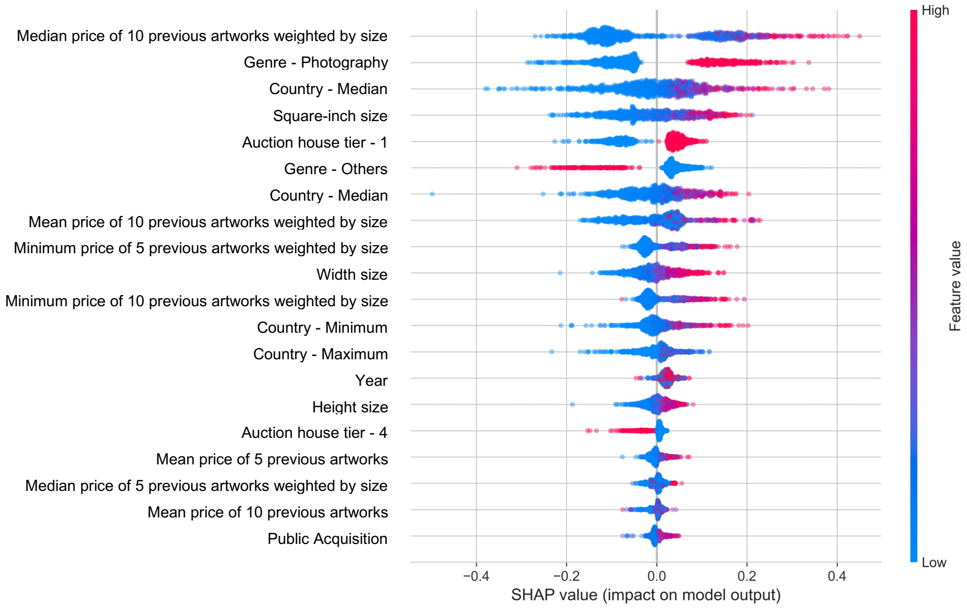

Figure S2. The SHAP values for the 20 most important features. All price-related features have a positive relationship with the output values — the estimated auction price of an artwork, which means that this feature pushes the model prediction higher. In contrast, the features including "Gender - Others" and "Auction house tier - 4" show a negative relationship with the estimated price meaning that having those features pushes the prediction lower.

example of the other class. For multiclass problems, we compute the AUC for each class in a "one versus rest" manner, then take the arithmetic mean of those values weighted by the number of examples of each class. The F1 score is the harmonic mean of precision and recall, which can be interpreted like an accuracy value which is less sensitive to class imbalance. We similarly compute the F1 score in a "one versus rest" manner for each class and take a weighted mean of the values.

Feature	Visual AUC	Visual F1	"Dummy" F1
Gender	0.502550	0.921839	0.925879
Education	0.495079	0.724212	0.724891
Elite School	0.502994	0.832800	0.846446
Genre	0.504080	0.254555	0.156049

Table S1. Prediction AUC and F1 scores for classification problems. Prediction is compared to a "dummy" classifier which always predicts the majority class (e.g. gender male, elite school, country USA). F1 score for multiclass problems are computed using weighted mean across each class. AUC scores are also computed using weighted mean, each class AUC is computed as self vs. other. The dummy classifier assigns random probability values and thus achieves exactly 0.5 AUC for each problem.
